# Nonmetastatic breast cancer patients subsequently developing second primary malignancy: A population‐based study

**DOI:** 10.1002/cam4.4351

**Published:** 2021-10-13

**Authors:** Shengnan Bao, Mengping Jiang, Xi Wang, Yijia Hua, Tianyu Zeng, Yiqi Yang, Fan Yang, Xueqi Yan, Chunxiao Sun, Mengzhu Yang, Ziyi Fu, Xiang Huang, Jun Li, Hao Wu, Wei Li, Jinhai Tang, Yongmei Yin

**Affiliations:** ^1^ Department of Oncology The First Affiliated Hospital of Nanjing Medical University Nanjing China; ^2^ The First Clinical College of Nanjing Medical University Nanjing China; ^3^ Department of General Surgery The First Affiliated Hospital of Nanjing Medical University Nanjing China; ^4^ Jiangsu Key Lab of Cancer Biomarkers, Prevention and Treatment Collaborative Innovation Center for Personalized Cancer Medicine Nanjing Medical University Nanjing China

**Keywords:** breast cancer, breast cancer‐specific survival, multiple primary cancer, nomogram, second primary cancer

## Abstract

**Background:**

With life span extending, breast cancer (BC) survivors may face the possibility of developing second primary cancer (SPC) and considerably shorten survivorship. However, little is known about multiple primary cancer (MPC) patients with nonmetastatic breast cancer as a first primary malignancy (BCFPM).

**Methods:**

Here, we retrospectively analyzed data on cancer survivors with BCFPM diagnosed between 2010 and 2015 from the Surveillance, Epidemiology, and End Results (SEER) database. The prognostic factors for breast cancer‐specific survival (BCSS) were ascertained by the stepwise regression analysis and a competing risk model, and were integrated to the establishment of prognostic nomogram, of which the accuracy was measured by the calibration curve and the concordance index (C‐index).

**Results:**

In total, 8616 patients were identified with 4.6% of 3‐year breast cancer‐ specific death (BCSD) and 8.6% of 5‐year BCSD. The most common SPC among BCFPM patients were female BC and lung cancer. Besides, the median latency time between BC and SPC was 22 months. At a ratio of 7:3, all patients were randomly categorized into a training cohort (*n* = 6032) and a validation cohort (*n* = 2584). By a proportional subdistribution hazards regression analysis, the following factors were considered to own independent prognostic abilities of BCSS: subtypes, grade, *T* classification, *N* classification, radiation, and sites of SPC. The nomogram could accurately predict 3‐year and 5‐year breast cancer‐associated survival of BCFPM patients with high internal and external validated C‐index, 0.715 (95% CI, 0.691–0.739), and 0.683 (95% CI, 0.642–0.724), respectively.

**Conclusions:**

BC survivors remained a high risk of developing SPC and considerably shortened survival time. In this study, a favorable nomogram was constructed to as a prediction model for 3‐year and 5‐year BCSS of BCFPM patients, largely intending to prolong the life of these patients by assisting clinicians to make individualized follow‐up plans.

## Graphical abstract

A remarkable number of second primary cancers (SPC) occur in breast cancer survivors and constitute an emerging subgroup of tumors, with a shortage of overall understanding and normative management. Here, we gathered a mass of retrospective data with precise definition of SPC and further explored the clinical characteristics and prognostic predictors of these patients, thus effectively avoiding the deviation caused by small sample and SPC misclassification.

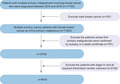

## 1 INTRODUCTION

Breast cancer (BC) is widely recognized as one of the most prevalent malignancies with highest morbidity for women worldwide. The encouraging fact comes that the prognosis of BC patients have greatly improved over the past decades, largely due to improvement of early detection and systematic treatment.^1^, ^2^, ^3^ Statistics from the American Cancer Society during 2009 through 2015 showed 5‐year survival rate for early‐stage BC survivors is over 90%, while approximately 30% for metastatic BC.^4^ Therefore, majority of BC survivors possessed a prolonged life expectancy, especially those without metastasis at the initial diagnosis, which made it possible to develop multiple primary cancers (MPC). In the United States, up to 7.9% of cancer survivors were diagnosed with more than one primary cancer, emphasizing the role of these patients with MPC cannot be ignored.^5^ Nonetheless, there are few researches on MPC, especially for MPC involving BC. In addition, it was evidence from large‐scale data that the clinical prognosis of BC survivors with MPC is not optimistic.^6^, ^7^

At present, TNM staging system, the most extensively used approach to evaluate survival outcomes, is not suitable for these patients who are related to special biological characteristics and complicated prognosis judgement. Thus, a dedicated clinical prognostic model for patients with MPC involving BC is urgently needed. Nomogram is obviously a better choice for this demand. It is a concrete graphical prediction tool that visually exhibits multiple independent prognostic variables to predict the clinical outcome we set.^8^, ^9^ To our knowledge, no published research has performed a nomogram for specific prediction on prognosis of BC survivors with MPC.

In this study, we focused on BC patients subsequently developing second primary cancer (SPC), because the vast majority of MPCs was dual primary cancer (DPC). We collected the patients’ available data in the Surveillance, Epidemiology, and End Results (SEER) database to make a deep understanding for this special group, including clinical information, the prevalent occurrence sites of the SPC and time duration between BC and SPC. In order to guide clinical strategies for patients, prognostic factors based on competing risk model were utilized to construct a systematic nomogram for accurate prediction in short‐term survival rates of MPC survivors with breast cancer as a first primary malignancy (BCFPM).

## 2 MATERIALS AND METHODS

### 2.1 Data source and study cohorts

**FIGURE 1:**
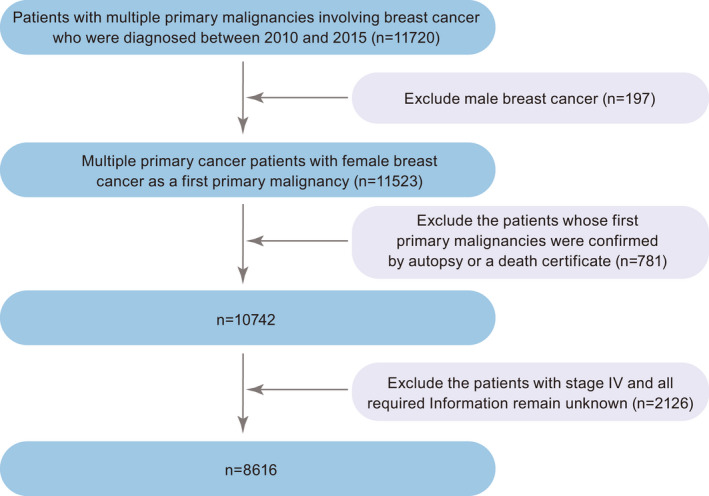
Flow diagram of the eligibility and exclusion criteria of the current study

A population‐based research was retrospectively operated with data from the SEER database, which incorporates national information on tumor samples from 18 large‐scale cancer registries and is open to public for cancer studies. From 2010 to 2015, a total of 8616 MPC sufferers with BCFPM were enrolled in this study, definitely including patients with two or more primary malignancies. Besides, we mainly focus on patients with nonmetastatic BC at the time of diagnosis, with consideration of low long‐term survival and complicated prognostic factors of metastatic (stage IV) BC. The particular clinical exclusion criteria were as follows: (1) male patients; (2) patients whose first primary malignancies were confirmed by autopsy or a death certificate; (3) patients with stage IV; (4) all required Information remain unknown. The elaborate patient screening process is exhibited in Figure 1.

### 2.2 Definition of SPC

A SPC is defined as independent cancer arising at least 2 months after the first primary malignancy, excluding recurrence, and metastasis. Clinically, the diagnosis of a SPC mainly depended on differential pathology findings from the primary and secondary lesions. In addition, the 2007 Multiple Primary and Histology Coding Rules from SEER were developed to assist in the classification of multiple primary malignancies, which were determined by pathology/cytology reports, sites of primary cancer, the interval between diagnoses, histology, and tumor behavior.^10^ It is of worth to note two key variables of identifying MPC in SEER, the “sequence number” of the MPC and “total number of in situ/malignant tumors for patient.”

### 2.3 Variable selection

The main variables we selected from SEER database included “race record,”, “Marital status at diagnosis,” “age at diagnosis,” “years of diagnosis,” “Breast subtype,” “ICO‐O‐3 Hist/behav, malignant,” “Derived AJCC Stage (7th ed),” “Rx Sumn‐‐Surg Prim Site (1998+),” “Radiation recode,” “Chemotherapy recode,” “Site recode B ICD‐O‐3/WHO 2008,” “COD to site recode,” “month since index,” (the time interval between two primary cancers), “SEER cause‐specific death classification,” “Survival months,” and “Vital status record (study cutoff used).” In order to avoid multicollinearity, we applied stepwise regression methods to gradually fit the model by adding or deleting covariables, and then determined eight candidate variables with strong predictive power.

### 2.4 Statistical analysis

Among patients with early diagnosed nonmetastatic BC, we should not overlook the significant impact of causes of death other than BC on this population.^11^ Thus, we selected breast cancer‐specific survival (BCSS) as our primary endpoint, whose definition turned to be the date from the diagnosis of the first primary malignancy (BC) until death due to BC. Moreover, competitive risk events were derived from non‐breast cancer‐specific causes of death. Simple random sampling was performed by the R software version 4.0.3, which contributed to the classification of a validation cohort and a training cohort with a ratio of 3 to 7. To achieve a more accurate estimated BCSS than Cox proportional hazard model, a proportional subdistribution hazards model, which could eventually calculate the survival rates of a particular outcome by considering competitive risk events, was utilized to ascertain prognostic factors associated with BCSS in the training cohort.^12^ Eight variables screened by stepwise method were considered in univariate analysis, and statistically significant covariates (*p* < 0.05 in the univariable analysis) were calculated on multivariate analysis. Then, the factors with statistical significance in multivariate analysis (*p* < 0.05) were incorporated into the final prognostic nomogram for predicting 3‐year and 5‐year BCSS using the R package “rms.” Both internal (derived from the training cohort) and external (derived from the validation cohort) validations were responsible for assessing the predictive precision of the models. The concordance index (C‐index) would reflect the predictive ability of the models and demonstrate its higher accuracy as the value lowered within a range of 0.5 to 1.0.^13^ The calibration curves were generated based on the comparison between the observed breast cancer‐related survival rates and the expected BCSS probabilities predicted by the nomogram. Statistical analyses and modeling were performed using R version 4.0.3 and its appropriate packages. Two sided *p* < 0.05 was considered significant.

## 3 RESULTS

### 3.1 Baseline clinical characteristics

**FIGURE 2:**
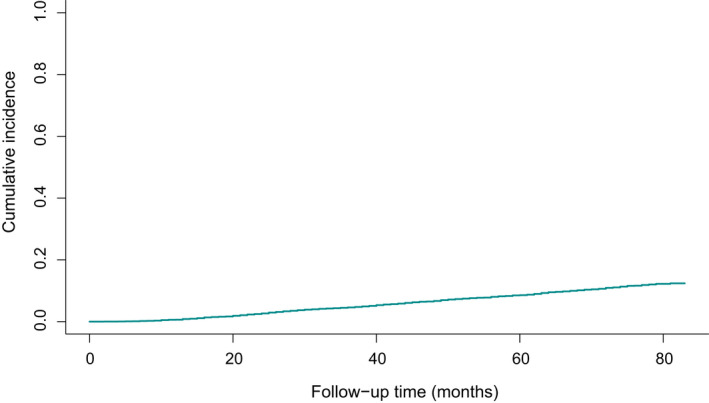
Estimates of overall cumulative incidence of breast cancer‐specific death, taking the other causes of death as a competing event

By primary screening, a total of 11720 patients with MPC involving BC diagnosed between 2010 and 2015 were abstracted from the SEER database. Of them, only 8616 MPC survivors with a prior nonmetastatic BC had information without missing so as to further analyze clinical and pathological characteristics (Figure 1). The cumulative incidence of the 3‐year, and 5‐year breast cancer‐ specific death for BCFPM patients were 4.6% and 8.6%, respectively, as displayed in Figure 2. According to a ratio of 7:3, those cases were categorized into two groups at random, a training cohort (*n* = 6032) and a validation cohort (*n* = 2584). The median follow‐up time of training cohort was 59 months. Their baseline demographic and clinicopathological characteristics are illustrated in Table 1. Besides, there were three groups in the entire study population classified by their age at the FPC (BC) diagnosis: young (aged ≤39 years), intermediate (aged 40–64 years), and elderly (aged ≥65 years). We set the cutoff points referred to previous published studies.^14^, ^15^ As shown in the table, the majority of patients in our analysis tended to be elderly, white, and married. In terms of molecular subtypes, 6606 (76.7%) patients presented with luminal A, followed by triple negative breast cancer (TNBC) patients with proportion of 11.3%. The other subtypes of luminal B and human epidermal growth factor receptor 2 (HER2) enriched accounted for 8.7%, 3.4%, respectively. Besides, a similar proportion of patients with either left breast tumor or right breast tumor were included in entire cohorts, whereas most of them had a tumor in the outer upper quadrant, at 34.1%. In terms of histologic type, ductal carcinoma is the largest group in our study. The patients diagnosed with T1, N0, or stage I, all over 50% of patients, had a higher frequency in both training and validation cohorts. Additionally, all patients underwent surgery, 60.6% of them suffer with lumpectomy, and 39.4% with mastectomy. Among them, patients receiving chemotherapy or radiation made up for 35.1% and 54.2%, respectively. In brief, above baseline variables were evenly distributed in both two cohorts.

**TABLE 1:** Demographic and clinicopathological characteristics of the training and validation cohorts

Variables	Entire cohort (*n* = 8616) (*N*, %)	Training cohort (*n* = 6032) (*N*, %)	Validation cohort (*n* = 2584) (*N*, %)
Age, years			
≤39	238 (2.8)	157 (2.6)	81 (3.1)
40–64	4016 (46.6)	2862 (47.4)	1154 (44.7)
≥65	4362 (50.6)	3013 (50.0)	1349 (52.2)
Race			
White	6987 (81.1)	4905 (81.3)	2082 (80.6)
Black	952 (11.0)	658 (10.9)	294 (11.4)
Other	677 (7.9)	469 (7.8)	208 (8.0)
Marital status			
Married	4665 (54.1)	3316 (55.0)	1349 (52.2)
Single	3951 (45.9)	2716 (45.0)	1235 (47.8)
Subtype			
Luminal A	6606 (76.7)	4637 (76.9)	1969 (76.2)
Luminal B	748 (8.7)	522 (8.7)	226 (8.7)
HER2 enriched	290 (3.4)	192 (3.2)	98 (3.8)
TNBC	972 (11.3)	681 (11.3)	291 (11.3)
Location			
Central portion	467 (5.4)	319 (5.3)	148 (5.7)
Upper‐inner quadrant	1113 (12.9)	759 (12.6)	354 (13.7)
Lower‐inner quadrant	490 (5.7)	344 (5.7)	146 (5.7)
Upper‐outer quadrant	2940 (34.1)	2098 (34.8)	842 (32.6)
Lower‐outer quadrant	596 (6.9)	408 (6.8)	188 (7.3)
Other	3010 (34.9)	2104 (34.9)	906 (35.1)
Histology			
Ductal	6320 (73.4)	4437 (73.6)	1883 (72.9)
Lobular	828 (9.6)	566 (9.4)	262 (10.1)
Other	1468 (17.0)	1029 (17.1)	439 (17.0)
Tumor grade			
Well	2173 (25.2)	1513 (25.1)	660 (25.5)
Moderately	3878 (45.0)	2715 (45.0)	1163 (45.0)
Poorly/undifferentiated	2565 (29.8)	1804 (29.9)	761 (29.5)
Laterality			
Left	4388 (50.9)	3080 (51.1)	1308 (50.6)
Right	4228 (49.1)	2952 (48.9)	1276 (49.4)
*T* classification			
1	5394 (62.6)	3771 (62.5)	1623 (62.8)
2	2554 (29.6)	1791 (29.7)	763 (29.5)
3/4	668 (7.8)	470 (7.8)	198 (7.7)
*N* classification			
0	6139 (71.3)	4282 (71.0)	1857 (71.9)
1	1771 (20.6)	1232 (20.4)	539 (20.9)
2–3	706 (8.2)	518 (8.6)	188 (7.3)
Stage			
I	4679 (54.3)	3274 (54.3)	1405 (54.4)
II	2945 (34.2)	2042 (33.9)	903 (34.9)
III	992 (11.5)	716 (11.9)	276 (10.7)
Surgery			
Lumpectomy	5217 (60.6)	3637 (60.3)	1580 (61.1)
Mastectomy	3399 (39.4)	2395 (39.7)	1004 (38.9)
Radiotherapy			
Yes	4673 (54.2)	3277 (54.3)	1396 (54.0)
No	3943 (45.8)	2755 (45.7)	1188 (46.0)
Chemotherapy			
Yes	3024 (35.1)	2114 (35.0)	910 (35.2)
No	5592 (64.9)	3918 (65.0)	1674 (64.8)
Location of SPC			
Female breast	2588 (30.0)	1805 (29.9)	783 (30.3)
Lung and bronchus	1153 (13.4)	820 (13.6)	333 (12.9)
Other	4875 (56.6)	3407 (56.5)	1468 (56.8)

Note

Abbreviations: HER2, human epidermal growth factor receptor 2; SPC, second primary cancer; TNBC, triple negative breast cancer.

### 3.2 The occurrence sites of SPC and the time interval between two primary cancers

Among all the cases, we mainly focused on MPC with BCFPM, so patients were included if involved with two or more primary malignancies. In total, 80 sites of SPC were observed between the 8616 BCFPM patients. As we can see from the Table 2, female breast turned out to be the most common sites of SPC, which reached up to 30%, followed by lung and bronchus (13.4%), corpus uteri (5.8%), thyroid (5.6%), respectively. The median interval time of the dual primary cancers mentioned above was 17, 25, 27, and 13 months. Besides, in the whole study cohorts, the median latency time of SPC diagnosis after BC was 22 months, and up to 50.1% of these observations was shown to be over 2 years. Breast cancer survivors had the shortest median interval of 13 months for undergoing another primary malignancy in the thyroid or the kidney, while the longest was 30 months for pancreatic cancer.

**TABLE 2:** Location of the second primary cancer (SPC) and median interval between two primary cancers

Location of SPC	*N* (%)	Median interval (months)
Total	8616 (100)	22
Female breast	2588 (30.0)	17
Lung and bronchus	1153 (13.4)	25
Corpus uteri	496 (5.8)	27
Thyroid	485 (5.6)	13
Melanoma of the skin	386 (4.5)	26
Kidney	284 (3.3)	13
Pancreas	279 (3.2)	30
Ovary	223 (2.6)	21
NHL‐nodal	194 (2.3)	23
Urinary bladder	190 (2.2)	27
Rectum	148 (1.7)	20
Acute myeloid leukemia	147 (1.7)	25
Cecum	142 (1.6)	21
Ascending colon	133 (1.5)	26
Sigmoid colon	129 (1.5)	20
Miscellaneous	124 (1.4)	28
Myeloma	118 (1.4)	24
Stomach	105 (1.2)	22
Others	1292 (15.0)	25

Note

Abbreviations: NHL, non‐Hodgkin's lymphoma; SPC, second primary cancer.

### 3.3 Independent prognostic factors for breast cancer‐specific survival

A univariate analysis based on competing risk model was performed on data of 6032 patients in the training cohort, with the purpose of exploring prognostic factors for BCSS of BCFPM patients. It was showed that marital status, subtypes, tumor grade, *T* classification, *N* classification, radiation, and location of SPC were closely related to survival of BCFPM patients. Meanwhile, these statistically significant factors were incorporated into the multivariate analysis as displayed in Table 3. The multivariate analyses revealed that subtypes, tumor grade, *T* classification, *N* classification, radiation, and location of SPC were independent prognostic factors for BCSS. Compared with patients aged ≤39 years, neither the intermediate patients nor the elderly patients were associated with survival outcomes (both *p* > 0.05). It is interesting to note that the prognosis of the single patients was worse than those married (SHR, 1.24; 95% CI, 1.04–1.48; *p* = 0.017). Unlike luminal patients, both HER2‐enriched patients (SHR, 1.77; 95% CI, 1.17–2.69; *p* = 0.007) and TNBC patients (SHR, 2.06; 95% CI, 1.64–2.58; *p* < 0.001) were found to have a higher risk of dying from BC. Besides, we can see from Table 3 that tumor grade was one of the independent prognostic factors of BCSS for BCFPM patients, statistics data indicated that patients diagnosed with well differentiated cancer had a better prognosis of BCSS than those with poorly or undifferentiated cancer (SHR, 3.15; 95% CI, 2.42–4.11; *p* < 0.001). Additionally, the higher the *T* classification or *N* classification was, the worse the overall survival prognosis was (all *p* < 0.001). Of note, the treatment of radiation significantly improved survival (SHR, 1.24; 95% CI, 1.04–1.48; *p* = 0.018). Remarkable differences of BCSS among diverse sites of SPC in MPC sufferers with BCFPM indeed existed in multivariate regression analysis.

**TABLE 3:** Breast cancer‐specific survival in univariate and multivariate analysis based on competing risk model in the training cohort

Risk factors	Univariate analysis SHR (95% CI)	*p* value	Multivariate analysis SHR (95% CI)	*p* value
Age, years				
≤39	Reference		Reference	
40–64	0.72 (0.43–1.20)	0.204		
≥65	0.72 (0.43–1.19)	0.198		
Marital status				
Married	Reference		Reference	
Single	1.24 (1.04–1.48)	0.017	1.15 (0.96–1.38)	0.126
Subtype				
Luminal A	Reference		Reference	
Luminal B	1.17 (0.85–1.61)	0.350	0.89 (0.64–1.24)	0.490
HER2 enriched	1.77 (1.17–2.69)	0.007	1.02 (0.67–1.56)	0.933
TNBC	2.06 (1.64–2.58)	<0.001	1.29 (1.01–1.66)	0.044
Tumor grade				
Well	Reference		Reference	
Moderately	1.35 (1.02–1.78)	0.036	1.09 (0.82–1.44)	0.567
Poorly/undifferentiated	3.15 (2.42–4.11)	<0.001	1.98 (1.47–2.65)	<0.001
*T* classification				
1	Reference		Reference	
2	2.29 (1.88–2.80)	<0.001	1.55 (1.25–1.92)	<0.001
3/4	5.13 (4.04–6.51)	<0.001	2.86 (2.17–3.75)	<0.001
*N* classification				
0	Reference		Reference	
1	2.21 (1.88–2.84)	<0.001	1.73 (1.40–2.15)	<0.001
2–3	3.90 (3.09–4.93)	<0.001	2.39 (1.83–3.12)	<0.001
Radiotherapy				
Yes	Reference		Reference	
No	1.24 (1.04–1.48)	0.018	1.31 (1.09–1.57)	0.004
Location of SPC				
Female breast	Reference		Reference	
Lung and bronchus	1.54 (1.20–1.98)	<0.001	1.77 (1.37–2.28)	<0.001
Other	0.86 (0.70–1.05)	0.144	0.93 (0.75–1.14)	0.484

Note

Abbreviations: CI, confidence interval; HER2, human epidermal growth factor receptor 2; SHR, subdistribution hazard ratio; SPC, second primary cancer; TNBC, triple negative breast cancer.

### 3.4 Development and validation of the prognosis nomogram

**FIGURE 3:**
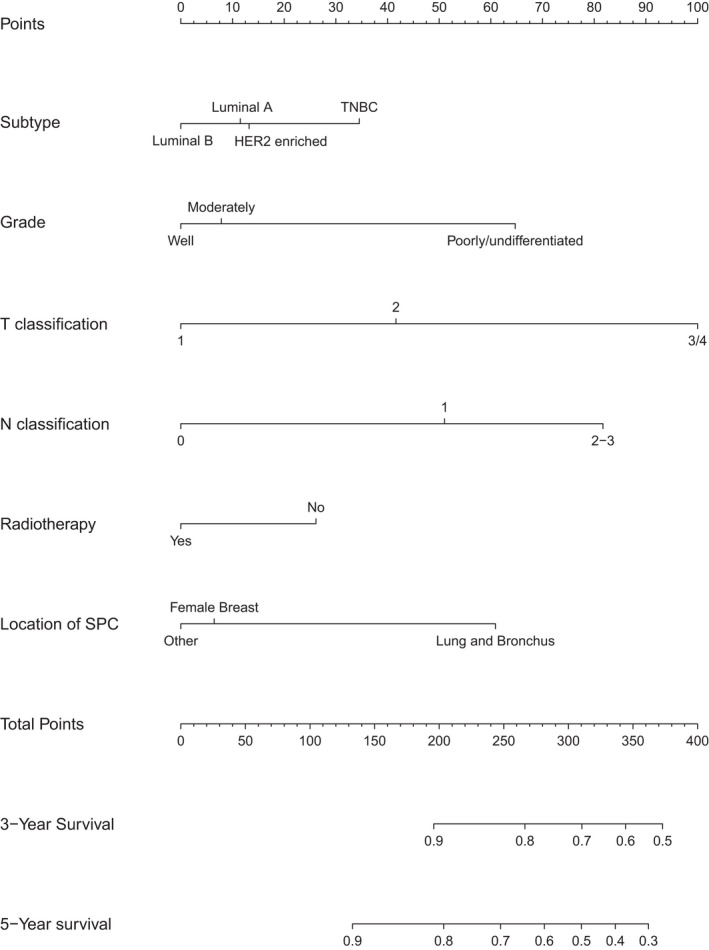
Prognostic nomogram of breast cancer‐specific survival (BCSS) in multiple primary cancer patients with nonmetastatic breast cancer as a first primary malignancy (BCFPM). Nomogram to predict 3‐year, and 5‐year BCSS of the patients. HER2, human epidermal growth factor receptor 2; TNBC, triple negative breast cancer; SPC, second primary cancer

**FIGURE 4:**
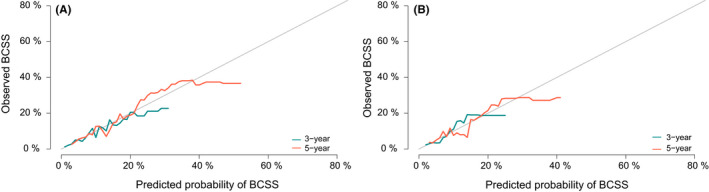
Internal and external validation of the nomogram. Calibration curves for 3‐year and 5‐year (A) breast cancer‐specific survival (BCSS) in the training cohort. Calibration curves for 3‐year and 5‐year (B) BCSS in the validation cohort. BCSS, breast cancer‐specific survival

On the basis of all the factors significantly associated with BCSS in the competing risk regression analysis, the estimated 3‐year, and 5‐year BCSS for individuals were predicted by a nomogram (Figure 3). It can see from the Figure 3 that *T* classification was the strongest contributor to prognosis, followed by *N* classification, tumor grade, sites of SPC, subtypes, and radiation. Different states of each clinical variable corresponded to different scores on the point scale. By summing the scores of each item, we could easily acquire the estimated probability of the 3‐year and 5‐year BCSS for BCFPM patients. The C‐index for the nomogram in the internal validation was 0.715 (95% CI, 0.691–0.739), and that of external validation was and 0.683 (95% CI, 0.642–0.724). As presented in Figure 4, the corresponding calibration curves of 3‐year, and 5‐year BCSS was drawn in each cohort, indicating a strong agreement between the nomogram‐predicted probabilities and realistic observation.

## 4 DISCUSSION

According to Global cancer statistics, BC has regarded as a main public health threat worldwide, with about 2.3 million new cases in 2020.^16^ Due to the development of early screening and the application of comprehensive treatment, the survival of BC patients has significantly improved, which naturally faces the possibility that these patients may develop the MPC. In recent years, patients with MPC have attracted researchers’ attention for its highly 18% incidence in cancer survivors based on SEER data.^17^ Previous studies indicated that MPC patients involving BC were related to worse outcomes than those with BC only.^7^, ^18^ Therefore, it is necessary for us to conduct in‐depth study on survival prognosis of this population. However, as far as we have concerned, there were no published literature to identity prognostic factors and construct a nomogram of nonmetastatic BC patients subsequently developing SPC. In our study, a retrospective analysis was operated to analyze 8616 MPC survivors with BCFPM diagnosed between 2010 and 2015 from the SEER database, aiming to evaluate prognosis of these patients and provide valuable recommendations for clinical treatment.

Notably, no clear standardized follow‐up plan is developed for MPC, especially for dual primary cancer survivors with prior BC. In general, the sites where the primary tumor tend to metastasize are of greater concern to clinicians during the follow‐up period, leading to inevitable neglect for other sites of developing secondary primary cancer. However, BC patients with SPC greatly shortened its survival time. Thus, with the purpose of designing effective follow‐up strategies, summarizing the common sites of SPC for patients with a history of BC is warranted. In this study, we clarified a total of 80 types of tumors as SPC among the 8616 BCFPM patients, and the most frequent sites of SPC are female breast, lung, corpus uteri, and thyroid, respectively. As similar to above results, a large‐scale data analysis in Korea showed that thyroid cancer finally proved to be the most common MPC for patients with prior BC, accounting for 48.5%, followed by gynecologic cancer, upper gastrointestinal cancer, colorectal cancer, and lung cancer.^6^ Those findings might remind clinicians that surveillance for female breast, lung, thyroid, and other common sites would also be preferable after the treatment of initial BC. In addition, we further explored the time interval between two primary cancers and revealed that median interval was 22 months, which partly address a matter of how often to follow‐ up after BC diagnosis. On the other hand, it is interesting to note that a few studies suggested treatment of primary cancers may increase the risk of developing SPC.^19^, ^20^ As observed in several studies, Hodgkin's lymphoma survivors were more likely to subsequently suffer BC, explained by the result of radiation therapy to the chest.^21^, ^22^

According to competing risk regression model, univariate and multivariate analyses were utilized to found that subtypes, tumor grade, *T* classification, *N* classification, radiation, and location of SPC appeared to be independent prognostic factors for BC survivors with SPC. Specifically, we demonstrated the relationship between dissatisfied survival outcomes and patients with MPC who were single at the time of BC diagnosis using SEER data. Besides, the result of our study showed that patients with TNBC in this group, compared to other molecular subtypes of BC, tended to be have a shorter survival time and similar finding could be observed in the general BC patients.^23^ This may be affected by a high degree of malignant behavior of patients with TNBC in biological characteristics and its resistance to most conventional treatments except chemotherapy.^24^, ^25^ In addition, we also found the unfavorable prognostic role of an advanced TN stage among the BCFPM patients, which was consistent with conventional wisdom that poor clinical features of BC were associated with poor clinical outcomes. Another finding was that patients with BCFPM receiving radiation had a higher propensity for longer survival time than those without treatment. That suggested that patients with BCFPM might benefit from the intervention of comprehensive and effective regimens for cancer survivors.

However, to date, less is known that the relationship between the prognosis of patients with BCFPM and SPC. Several studies have declared that the definite diagnosis of SPC after BC was regarded as an independent risk factor and led to significantly shorten the life span of patients.^26^, ^27^ In our study, we not only confirmed that subsequent primary cancer indeed worsened survivorship in BC survivors using SEER data, but also further revealed that the survival of patients with BCFPM varied depending on occurrence sites of SPC. Among several groups of different locations of SPC, the adverse impact on survival of patients with second primary lung cancer was the most obvious, while influence on the patients who developed dual primary BC was relatively minimal. Despite of this, there were a few controversial findings from other studies. A retrospective analysis in Korea reported that a relatively good prognosis occurred in patients with thyroid cancer as SPC, but the prognosis of prior BC had nothing to do with it.^6^ Conversely, another research found a poorer prognosis of patients who developed thyroid cancer after BC, compared to those with BC alone.^7^ In this regard, it may be caused by differences in data sources and statistical analysis methods. Interestingly, in the case of second primary lung cancer, previous studies indicated that a BC history may result in better prognosis for non‐small cell lung cancer (NSCLC) patients, which we think may be due to the effective and varied treatment of BC.^18^ In a word, more further researches and statistical evidence are required to explore the influence of subsequent primary cancer on survival rate of patients with BCFPM.

Our research has the following advantages. First of all, large‐scale information from the SEER database is one of the highlights of our study, including 8616 BCFPM patients among 11720 patients with MPC involving BC. Moreover, the SEER database has high data accuracy, and the default histological criteria for defining MPC make the impact of misclassification errors in the study minimal.^10^, ^18^ Furthermore, to our knowledge, it is the first attempt to use nomogram to construct a clinical prediction model for survival time of BCFPM patients by calculating the 3‐year or 5‐year BCSS as the high C‐index showing good consistency and ideal predictive value. Instead of Cox proportional hazard model, the utilization of the competing risk model strengthens the credibility and rationality of our research. Therefore, we had an unbiased estimate of BCSS to reflect a more realistic survivorship of nonmetastatic BC patients in the presence of competing risk. Finally, no matter the site distribution of SPC and the interval time between two primary cancers, or the nomogram we established for evaluation of patient survival prognosis, all these initial findings were inclined to facilitate clinicians to deepen the understanding of MPC patients with a prior BC for better clinical decision‐making.

Certainly, some shortcomings do exist in the present study. The first disadvantage is the limitation of the collected data in the SEER database. In addition to the lack of detailed chemotherapy regimens, there is no complete information on family history, lifestyle characteristics, Body Mass Index (BMI), tumor markers, and other potential risk factors for BCFPM patients. Second, we have to admit the selection bias of the retrospective study despite the valuable ability of proportional subdistribution hazards model to adjust confounding factors. Third, as researches on prognosis of MPC patients involving BC is still not abundant, the published findings only serve as recommendations for clinicians instead of consensuses. Therefore, there is imperious demands to develop further prospective studies to validate our findings.

## 5 CONCLUSION

In conclusion, we indicated that the most common site of SPC after BC was female breast, followed by lung, corpus uteri, thyroid, and so on. Additionally, the median interval time between two primary cancers in our study was 22 months, and second primary BC was the shortest of 13 months. Furthermore, our study revealed the prognosis of nonmetastatic BC patients subsequently developing SPC determined by subtypes, tumor grade, *T* classification, *N* classification, radiation, and location of SPC. A clinically useful competing risk nomogram was constructed to predict the 3‐year and 5‐year BCSS for BCFPM patients. Our findings could be helpful for clinicians to make individualized follow‐up plans for these patients, which is expected to prolong the life of patients by early detection and treatment. However, more effort on studies about screening strategies for SPC patients after BC are needed.

## CONFLICTS OF INTEREST

The authors have declared that no competing interest exists.

## ETHICAL APPROVAL STATEMENT

This study was exempted by the institutional ethics committee of The First Affiliated Hospital with Nanjing Medical University because our data were from the SEER database, which is open to the public. All procedures performed in studies involving human participants were in accordance with the Helsinki declaration and its later amendments or comparable ethical standards.

## Data Availability

A population‐based research was retrospectively operated with data from the SEER database, which incorporates national information on tumor samples from 18 large‐scale cancer registries and is open to public for cancer studies (https://seer.cancer.gov/).
